# Hypoxia causes woodlice (*Porcellio scaber*) to select lower temperatures and impairs their thermal performance and heat tolerance

**DOI:** 10.1371/journal.pone.0220647

**Published:** 2019-08-01

**Authors:** Andrzej Antoł, Wiktoria Rojek, Sanjeev Singh, Damian Piekarski, Marcin Czarnoleski

**Affiliations:** Institute of Environmental Sciences, Jagiellonian University, ul. Gronostajowa, Kraków, Poland; Universidad de la Republica Uruguay, URUGUAY

## Abstract

Environmental temperatures and oxygen availability are important for the balance between oxygen supply and demand. Terrestrial organisms are generally perceived to be less limited by access to oxygen than their aquatic counterparts. Nevertheless, even terrestrial environments can be deficient in oxygen, especially for organisms occurring in soil, litter, wood, rotten fruit or at high elevations. While isopods are the best adapted to a terrestrial lifestyle among crustaceans, many species, including woodlice, occupy environmental gradients of temperature and oxygen. To investigate whether mismatches between oxygen supply and demand can result in a loss of performance in a terrestrial organism, we studied the effects of atmospheric oxygen concentration on the thermal performance of the common rough woodlouse (*Porcellio scaber*). We compared the thermal preference, thermal sensitivity of running speed, and tolerance to extreme temperatures of woodlice exposed to one of two oxygen concentrations (21% - normoxia, 7% - hypoxia). Under hypoxia, *P*. *scaber* preferred microhabitats with temperatures that were on average 3°C lower than those preferred under normoxia. The running speed tended to reach its maximum at a lower temperature under hypoxia than under normoxia (25.13°C vs 28.87°C, respectively, although p was equal to 0.09), and normoxic woodlice ran approximately 1.5-fold faster than hypoxic woodlice at the point of maximum speed. Heat tolerance was significantly lower under hypoxia (38.9°C) than under normoxia (40.7°C), but there was no difference in cold tolerance (5.81°C under normoxia and 5.44°C under hypoxia). Overall, our results indicate that environmental gradients of temperature and oxygen may shape the physiological performance of terrestrial ectotherms, likely via their effects on the balance between oxygen supply and demand, which may have fitness consequences for these organisms in nature.

## Introduction

Isopods appear to be the most successful land colonizers of all crustaceans [1], and it is estimated that their terrestrial lifestyle evolved independently at least two times [2]. They evolved number of land adaptations, such as pleopodal lungs, a water-conducting system and aggregation and conglobation behaviours [1]. It remains unclear which selective factors drove this evolutionary transition, but a lower risk of predation and access to oxygen and/or food are likely candidates [3,4]. On the other hand, terrestrial environments can impose new challenges to land colonizers, such as stronger thermal fluctuations combined with an increased risk of desiccation and overheating. Interestingly, the colonization of land by isopods ca. 300 Mya [5,6] coincided with peak levels of atmospheric oxygen, followed by a large (50%) and rapid decrease in the atmospheric oxygen content [6,7]. Therefore, isopod lineages evolving a terrestrial lifestyle experienced dramatic shifts in oxygen availability throughout their evolutionary history: from the relatively low oxygen availability in aquatic environments [8] to the initially high but later decreased oxygen availability on land. Contemporary isopods often inhabit microenvironments in close contact with decomposing organic matter, which are characterized by a lower oxygen supply than the atmosphere. For example, the concentration of oxygen in wet, decaying beech logs (a potential habitat for isopods) can be reduced 40-fold, reaching concentrations as low as 0.5% [9]. Some isopods inhabit periodically submerged sand burrows in intertidal zones, where they can be exposed to hypoxic conditions [10]. Isopods were also reported to inhabit altitudes reaching 4725 m a.s.l. [11], where oxygen pressure can drop to 60% of the pressure at sea level [12].

The oxygen supply is regarded as an important environmental characteristic that has strong fitness consequences because it impacts a myriad of organismal performance metrics, ranging from, e.g., consumption [13], metabolism [10] and growth [14] to behavioural reactions to predators [15]. Nevertheless, an effect of oxygen supply on organismal performance is relative because it strongly depends on the metabolic demand, which is largely determined by the physical and physiological work and body temperature [14]. In principle, the performance of ectotherms is stimulated by warm environments, but as the environmental temperatures approach critical values, their performance rapidly deteriorates, which is ultimately followed by death [16]. Much of the research in the area of thermal performance of ectotherms focuses on the critical temperatures that suppress performance and survival [17–19]. The ability to cope with thermal limits can be governed by the thermal sensitivity of molecules, mainly enzymes [20] and phospholipids [21], but it is also hypothesized to be linked to a temperature-driven imbalance between metabolic supply and demand, which would lead to insufficient oxygen delivered to tissue under thermal extremes [22]. Indeed, upper thermal limits seem to be reduced by hypoxia in many aquatic organisms [23–25], but this phenomenon has been far less studied in terrestrial organisms, which are generally regarded as less often exposed to oxygen deficiency under natural conditions [26]. Nevertheless, some data on lizards [27], insects [28,29] and terrestrial isopods [30,31] suggest that the oxygen supply can affect thermal performance, even in land-dwelling ectotherms, although there is evidence suggesting that thermal limits in terrestrial organisms are affected by only severe hypoxia [32]. The temperature-driven mismatch between oxygen supply and demand also seems to scale up to the level of thermal dependence of the life histories of ectotherms. The thermal environment directly affects ectotherms’ fitness [16] by governing physiological rates [33], predation [34] and mobility [35]. Puzzlingly, most ectotherms mature earlier and reach smaller adult sizes in warmer environments – a pattern often called the temperature-size rule [20]. There is theoretical and empirical evidence that this puzzling pattern is governed by resource allocation to growth and reproduction, which adaptively responds to thermal changes in metabolic supply and demand for oxygen [14,36–39].

Addressing the oxygen-dependent thermal performance of isopods, we performed laboratory experiments on the common rough woodlouse (*Porcellio scaber*). This species of isopod naturally occurs in Europe, excluding south-eastern Europe, and has been introduced to many other continents, such as North America and Australia [40]. *P*. *scaber* woodlice inhabit decaying leaf litter and logs, so they should naturally occupy an array of microhabitats that differ in thermal and oxygen conditions. To assess the combined effects of the thermal and oxygen environments on performance, we first performed a choice experiment in which we exposed the studied woodlice to a wide thermal gradient, testing whether their thermal preference undergoes changes with the level of atmospheric oxygen. We hypothesized that woodlice would select cooler microhabitats under hypoxia, decreasing their oxygen demand [41] and/or increasing oxygen affinity of the haemolymph [42,43]. Then, we studied the thermal sensitivity of running speed and thermal physiological limits, testing how oxygen conditions shape these characteristics. Given that a limited access to environmental oxygen should impede aerobic capacity, we predicted that low-oxygen conditions would decrease the maximum level of performance and shift this maximum towards lower temperatures, where decreased demand for oxygen [41] would meet increased oxygen supply associated with the improved oxygen affinity of the haemolymph [42,43]. Since the evidence for oxygen limitation is stronger for heat tolerance than for cold tolerance [44], we expected that the hypoxic woodlice would lower their heat tolerance but tolerate cold extrema equally as well as normoxic woodlice. It is because hypoxia should impose limits especially in combination with increased metabolic demand caused by higher body temperature.

## Material and methods

**Fig 1:**
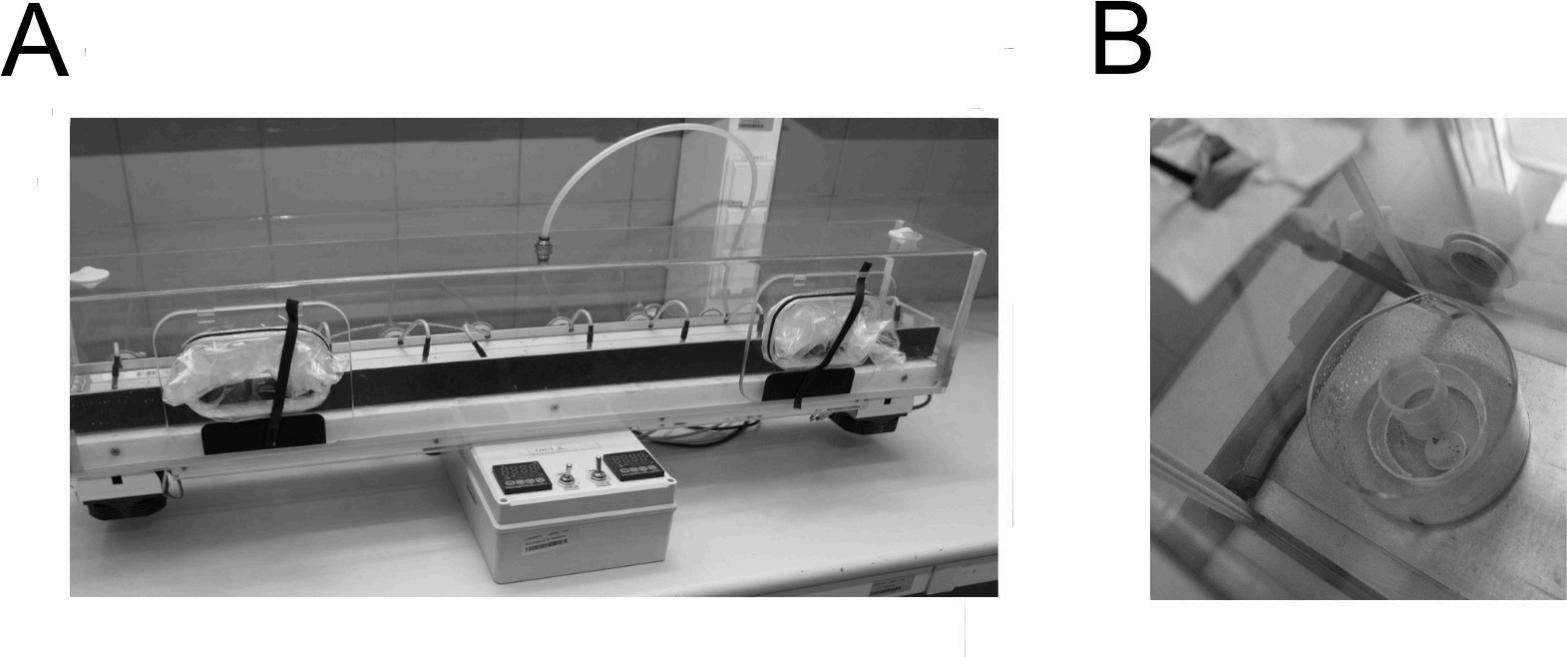
**A:** Thermal platforms made of a metal bar with two Peltier modules on either side were used to measure thermal preference, thermal performance and critical temperatures. The whole system covered with a transparent chamber that allowed for the regulation of oxygen conditions under the cover. **B:** An arena used to measure the locomotor activity of woodlice. A larger container was located on the metal platform just above the Peltier module. The bottom of the container was covered with moist sand. The arena in which animals ran was limited by a plastic circle. The temperature logger and the cylinder in which the animal was acclimated were placed inside the circle. The arena brush, which could be manipulated from the outside, is located above.

For this study, we collected *P*. *scaber* in late summer from two monastery gardens (50.059179 N, 19.93604 E; 50.065117 N, 19.931388 E) and from one old backyard (50.070957 N, 19.939061 E) in the vicinity of the Old City of Kraków (Poland). The species is not under protection, and we obtained permission from the landowners to collect it. The animals were maintained at the Institute of Environmental Sciences (Kraków) in a climatically controlled room set to 20°C and with a 12D:12L photoperiod. Once per week, the animals were provided water and a dry leaf mixture consisting mostly of alder (*Alnus glutinosa*) and ash (*Fraxinus excelsior*). Our experiments included both sexes, and prior to each experiment, the animals were weighed to the nearest 0.001 mg (XP26, Mettler Toledo, Greifensee, Switzerland). In all experiments, we used three thermal platforms connected to an oxygen regulation system (Fig 1). The platforms were built from a one-metre-long metal bar with two Peltier modules on each side (BIOSPEKT, Kraków, Poland). The modules enabled us to either heat or cool one of the two sides of the bar to obtain a thermal gradient (Experiment 1) or generate a desired temperature for studying thermal performance (Experiments 2 and 3). A platform was enclosed in a transparent Plexiglas cover (YETI, Agencja Reklamy, Kryspinów, Poland), which provided the tested animals with the experimental oxygen concentrations. The concentration of oxygen was continuously monitored by a fuel cell (Sable Systems International, Las Vegas, NV, USA), and hypoxia (7% O_2_) was maintained by adding nitrogen (Air Products Sp. z o.o., Kraków, Poland) with a Roxy-4 controller (Sable Systems International), which added gas according to the demand needed to decrease the oxygen level inside the chamber. We used external air to create normoxic conditions. The woodlice involved in all experimental essays were tested on a layer of moist sand (160 ml of water per 500 ml of dry sand; hereafter, moist sand), which provided a semi-natural substrate for the tested animals.

### Experiment 1: Thermal preferences

To measure thermal preference, woodlice were placed on a gradient of temperatures generated by the thermal platforms, and each individual woodlouse could freely choose thermal conditions. The Peltier modules on the two ends of each thermal platform were set to either 12°C or 45°C to generate a wide thermal gradient. An 8-cm-wide aluminium u-shaped profile was placed on the platform, and its bottom was covered with moist sand. The local temperatures were recorded in 30-minute intervals (to the nearest 0.1°C) every 10 cm along the thermal gradient with the help of thermocouples placed in the sand and connected to a computer. After each session, the sand was replaced by fresh sand, which ensured comparable humidity conditions between sessions and allowed us to avoid possible influences of cues left by previously tested animals. During each session, we measured the preferences of three animals, but to ensure independent measures, each animal was placed individually into a narrow corridor. The corridors were produced from 1-cm-wide u-shaped aluminium profiles that ran along the platform. The top of the profile was perforated to ensure air access for the animal. Prior to the measurements, each animal was placed in a random position along the thermal gradient (we used a random number generator) and covered with the profile used to create the corridor. Each measurement session started at 7 p.m. and lasted 12 h. During the sessions, soft light (10% brightness) was provided in the climatically controlled room. In the morning on the next day, the positions of the animals in each corridor were recorded. We measured the distance from each animal to the two nearest thermocouples, which was used to calculate the local temperature at the animal’s position (considered the preferred temperature). The calculation assumed a linear change in the temperatures between the two points where the temperature was measured. In total, we tested 79 animals, both males (34 individuals) and females (45 individuals). On average, each tested animal enclosed in a corridor was exposed to an effective gradient of temperatures that ranged from 16.41°C to 36.28°C (as measured directly in the sand at the two ends of the thermal gradient); along this gradient, the temperature changed at an average rate of 0.21°C per 1 cm.

### Experiment 2: Thermal performance

To measure the oxygen-dependent thermal performance, the animals were forced to run around a circular glass arena, and we measured their average speed at different temperatures and oxygen levels. The arena was built from a glass container (80 mm in diameter) with a small plastic cylinder in the middle, which created a circular corridor for performing the running assays (Fig 1). Inside this plastic cylinder, another cylinder was placed in which the animal was acclimated. To provide the running animals with a semi-natural substrate, the bottom of the arena was covered with a thin layer of moist sand. During measurements, the arena was placed on the thermal platform, immediately above one of the two Peltier modules, which was set to one of 13 different temperatures: 8, 11, 14, 17, 20, 23, 26, 29, 32, 35, 38, 41, or 45°C. Temperatures were recorded to the nearest 0.1°C by an iButton (Maxim/Dallas Semiconductor, San Jose, CA, USA) placed in the middle of the arena. The measurements were performed under either hypoxia or normoxia.

Prior to testing, an animal was briefly habituated to the test conditions for 15 minutes in a narrow plastic cylinder located in the middle of the arena used for running tests. During measurements, animals were induced to run by touching the posterior part of the body with a small brush. The brush was manipulated by hand from the outside of the cover that surrounded the thermal platform. To reach the animal with the brush from the outside of the arena without changing the conditions under the cover, the brush penetrated the cover via a flexible material that allowed the entry point to be sealed (Fig 1). The tested animal was placed at a starting point (a line painted on the walls of the arena) and then forced to run. After each lap in the arena (indicated by passing the starting point), the time was recorded on a computer with the help of the estopwatch.net program. The running assay lasted for up to 15 minutes. If an animal did not react to the three touches of the brush during the assay, it was assumed to be exhausted, and the test was ended. For the analysis, we calculated the mean time required to complete one lap in the arena. For logistical reasons, the experiment was run in two fully balanced rounds. For each temperature and oxygen combination, we tested 4 animals (2 males and 2 females). If the temperature measured by the iButton during the assay deviated by 2°C or more from the desired temperature, the test at that temperature was repeated with other animals, but both results were included in the analysis. In total, we tested 114 animals, both males (56 individuals) and females (58 individuals).

### Experiment 3: Critical temperatures

To measure oxygen-dependent critical temperatures, we observed changes in the capacity of woodlice to control their body position during exposure to gradual changes (either an increase or a decrease) in temperatures under hypoxia and normoxia. The tested animals were placed in a small plastic container with a metal bottom, and the container was placed on the thermal platform above one of the two Peltier modules. Note that the oxygen conditions were controlled in the same way as in Experiments 1 and 2. The bottom surface of the container was divided into two halves, and each half was dedicated to one animal (two animals were tested simultaneously). To provide a semi-natural substrate for the animals, the bottom of the container was covered with a thin layer of moist sand. The temperature during measurements was recorded to the nearest 0.01°C by a fast-response thermocouple thermometer (HD 2128.2, Delta OHM, Caselle di Selvazzano, Italy) connected to a computer. The upper critical temperature (CT_max_) was measured by placing an animal in the test container, allowing the sand temperature to reach 35°C, and then exposing the animal to a steady increase in temperature at a rate of 0.5°C per minute. After each 0.5°C increase, we used a brush to turn the animal over onto its back. If the animal did not regain its position within 30 seconds, the temperature was considered the CT_max_. For the analysis, the mean of the temperatures recorded every second over this 30-second interval was used as the measure of CT_max_. In total, we tested 21 animals, both males (10 individuals) and females (11 individuals). The critical minimum temperatures (CT_min_) were measured by placing an animal in a test container when the sand temperature reached 3.5°C. After the animal was put into a chill-induced coma, which was confirmed by turning the animals over with a brush and checking if they regained their position within one minute, we switched off the Peltier module on the thermal platform, allowing the temperature to steadily increase at a rate of 0.5°C per minute. This rate was determined prior to the measurements. The temperature at which the animal regained its normal position was considered the CT_min_. In total, we tested 24 animals, both males (12 individuals) and females (12 individuals).

### Statistical analysis

The analysis was performed in R 3.4.1 software [45] with the help of the nlme [46], ggplot2 [47] and effects [48] packages. The data on preferred temperatures and critical temperatures (CT_max_ and CT_min_) were analysed with a general linear model (GLM) including sex and oxygen conditions (with an interaction term) as grouping predictors and body mass as a covariate. The running speed data were analysed with a nonlinear mixed model (nlme function). The fixed parts of the model were sex as a grouping predictor and temperature and body mass (without interactions) as numeric predictors. Following Lachenicht et al. [49], we assumed that the thermal dependence of the running speed, our thermal performance curve, took the shape of a 3^rd^-order polynomial function. The random parts of the model were the experimental round as well as the random estimates of our thermal performance curve parameters. To test if the effect of the experimental round was significant, we ran a similar model without the random effect (using the lm function). We compared the Akaike information criterion (AIC) values of these models and chose the best model as that with the lowest AIC.

To examine whether normoxia and hypoxia were characterized by different maximal performances (MPs) and temperatures at which these maxima were achieved (T_MP_), we designed a simplified version of the GLM that considered only oxygen and temperature as fixed factors. In the first step, we estimated parameters of the fitted thermal performance curve (3^rd^ polynomial) for normoxia and for hypoxia. After differentiation of these functions, we computed the point at which the first derivative of each function reached zero, which corresponded to finding the MP and T_MP_. In the second step, we calculated differences in MP and T_MP_ between the oxygen treatments. To test whether these differences were statistically significant, we used an approximate randomization test [50], which compared the observed differences in MP and in T_MP_ with a distribution of randomly generated differences in MP and in T_MP_. An observed difference was regarded significant when its value occurred among 5% of the rarest randomly generated values. The distribution of randomly generated differences was produced via 10000 randomizations. Each randomization involved i) pooling the data on the temperature dependence of running speed in our two oxygen treatments, ii) randomly re-assigning these data to our oxygen treatments, iii) computing the MP and T_MP_ for the two (normoxia and hypoxia) randomly generated thermal performance curves (we used the same statistical and mathematical tools that were used to calculate the observed values of MP and T_MP_), and iv) computing the difference in MP and in T_MP_ between the two randomly generated curves.

Above 37.59°C, some animals died during our performance tests, and the data for these cases were not used in the computation of thermal performance curves. Nevertheless, we decided to further explore these mortality data in a separate analysis that addressed whether the risk of mortality during our tests depended on oxygen conditions. We used a chi-square test that compared the proportion of dead animals between the normoxia and hypoxia conditions in tests carried out above 37.59°C.

## Results

**Fig 2:**
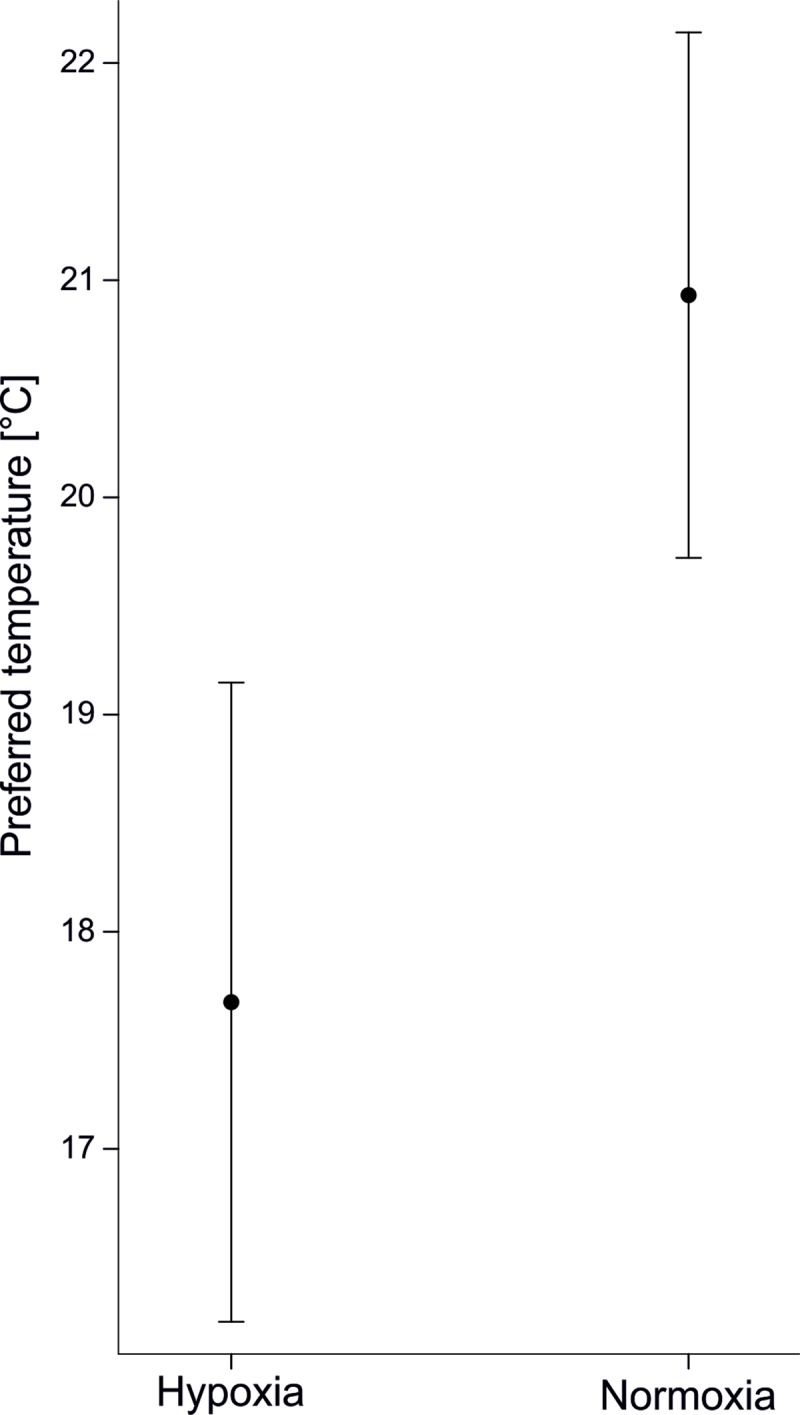
Common rough woodlice preferred lowertemperatures when exposed to oxygen deficiency (p = 0.001). Means ± 95% CIs from the general linear model with body mass as a covariate and sex as an additional factor.

Compared to woodlice exposed to normoxic conditions, woodlice exposed to hypoxia selected microsites with lower temperatures (F_1,74_ = 11.66, p = 0.001); on average, the preferred temperatures under hypoxia and normoxia were 17.68°C and 20.93°C (Fig 2), respectively. The sex of woodlice (F_1,74_ = 0.50, p = 0.48), the sex x oxygen interaction (F_1,74_ = 1, p = 0.32), and the body mass of woodlice (F_1,74_ = 0.31, p = 0.58) did not affect temperature selection.

**Fig 3:**
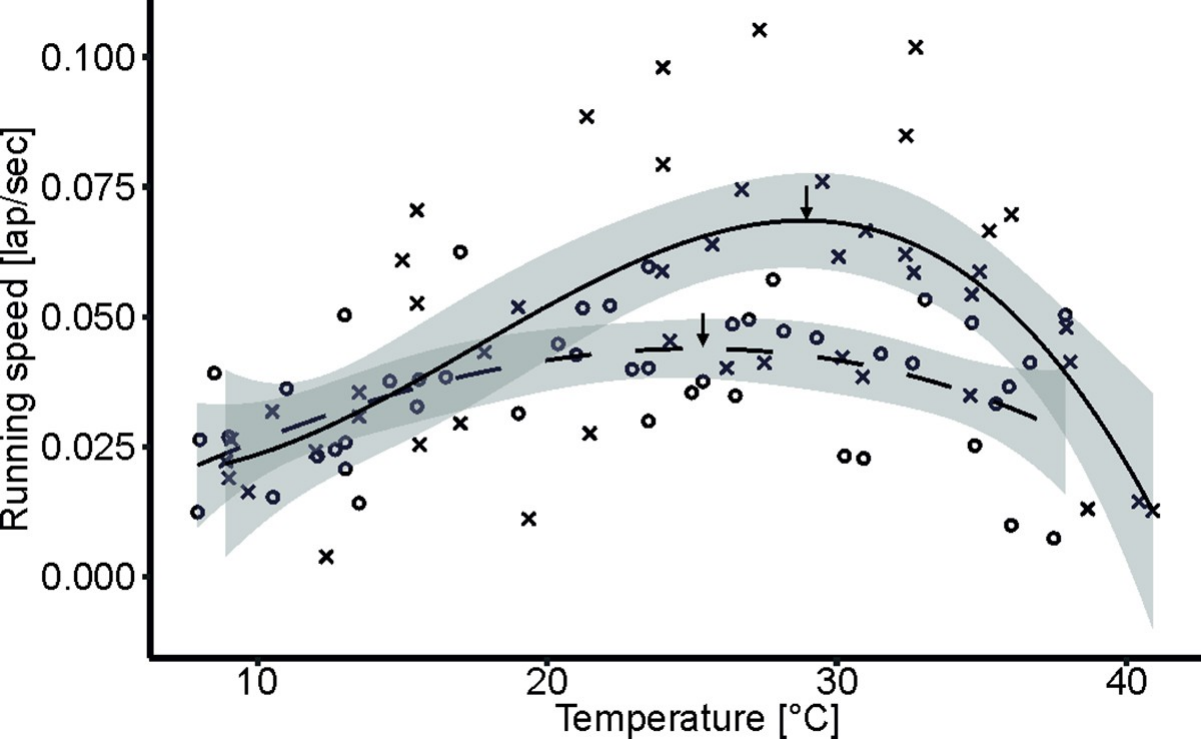
The thermal performance of common rough woodlice was lower under hypoxia (dashed line and circles) than under normoxia (solid line and x shapes). The cubic regression line under hypoxia is described by the formula y = -0.0003+0.003x-0.00003x^2^ – 0.0000007x^3^, and that under normoxia is described by y = 0.03-0.004x+0.0004x^2^ -0.000008x^3^. Arrows depict functional maxima that define the maximum performance (MP) and the temperature at which this maximum was achieved (T_MP_). Shaded areas show the 95% CIs of the fitted curves.

**Fig 4:**
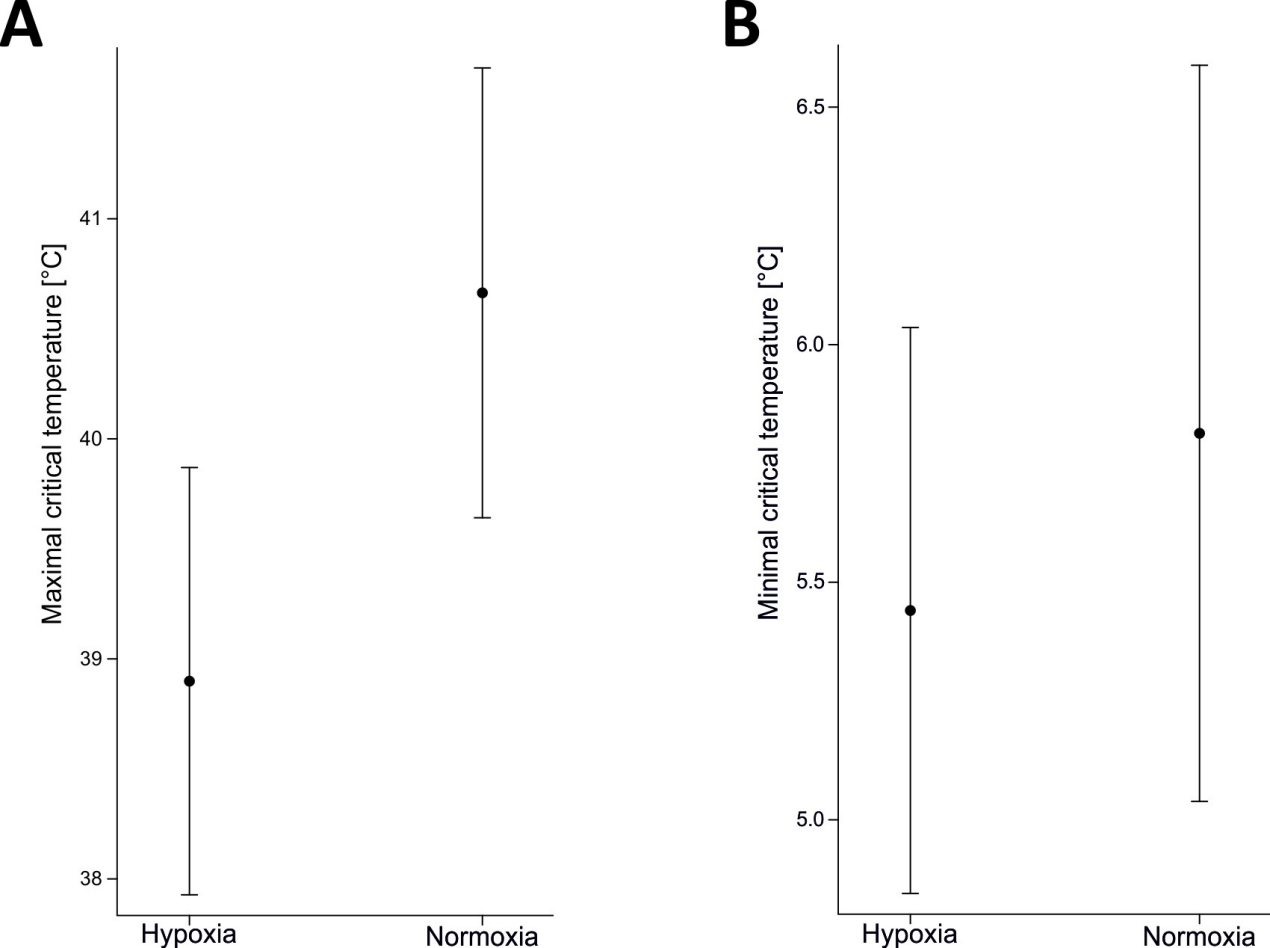
**A**: Common rough woodlice tolerated hotter environments under normoxia than under hypoxia, and they met their upper thermal limits at lower temperatures under hypoxic conditions than under normoxic conditions (p = 0.02). Means ± 95% CIs are from the general linear model with body mass as a covariate and sex as an additional factor. **B:** Oxygen availability had no effect on the tolerance of common rough woodlice to low temperatures (p = 0.42). Means ± 95% CIs are from the general linear model with body mass as a covariate and sex as an additional factor.

In the analysis of the thermal sensitivity of running speed, the model without a random effect had a lower AIC score than that with the random effect; consequently, the results of this best model are presented here. Sex (F_1,84_ = 1.65, p = 0.20) and body mass (F_1,84_ = 0.11, p = 0.75) did not affect the performance of woodlice. Two parameters of the performance curve differed between hypoxia and normoxia (intercept: F_1,84_ = 14.47, p = 0.001; linear coefficient: F_1,84_ = 6.3, p = 0.01), and two were not different (quadratic coefficient: F_1,84_ = 2.21, p = 0.14; cubic coefficient: F_1,84_ = 1.83, p = 0.18). The temperature at which maximal performance was reached (T_MP_) was 25.12°C under hypoxia and 28.87°C under normoxia (Fig 3). Our randomization test showed that the difference between these temperatures was almost significant (p = 0.09; S1 Fig). The maximal performance at these temperatures (MP) was 0.044 laps per sec under hypoxia and 0.069 laps per sec under normoxia, and this difference was significant (p<0.001; S2 Fig). Compared to normoxic conditions, hypoxia resulted in an increased proportion of animals that did not survive the performance tests at temperatures above 37.59°C (χ^2^ = 6.13, p = 0.01), indicating that hypoxic conditions lowered the tolerance of woodlice to high temperatures. In the analysis of critical temperatures, we found that animals exposed to hypoxia had a lower CT_max_ (38.90°C) than animals exposed to normoxia (40.66°C) (F_1,16_ = 6.41, p = 0.02, Fig 4). Sex (F_1,16_ = 0.02, p = 0.88), the sex x oxygen interaction (F_1,16_ = 2.50, p = 0.13), and body mass (F_1,16_ = 0.07, p = 0.80) did not affect CT_max_. In contrast, the value of CT_min_ (5.81°C under normoxia and 5.44°C under hypoxia, Fig 4) was not affected by oxygen (F_1,19_ = 0.67, p = 0.42), sex (F_1,19_ = 0.09, p = 0.76), the sex x oxygen interaction (F_1,19_ = 0.67, p = 0.42) or body mass (F_1,19_ = 0.15, p = 0.70).

## Discussion

Our experimental results indicate that when exposed to hypoxic conditions, *P*. *scaber* chooses to occupy microsites with low temperatures. In nature, woodlice inhabit sites with different oxygen conditions [10], so our evidence suggests that isopods exposed to poor oxygen conditions, e.g., those due to rapid decomposition or high altitude, might prefer to stay in cooler sites. The links between oxygen availability and preferred temperatures have rarely been studied, but the available evidence shows that oxygen deprivation also decreases preferred temperatures in some species of protists [42], fish [43,51], amphibians, reptiles [41], and even mammals [42]. This effect of hypoxia on the choice of thermal environments can be explained by a number of different mechanisms, depending on the biology and ecology of the tested species. For example, in ectotherms, selecting cooler microhabitats will lower physiological rates and hence oxygen demand [41]. In animals with oxygen-binding metalloproteins, such as the haemocyanin in isopods, lower body temperature increases blood affinity to oxygen [42,43]. In actively breathing animals, lower temperatures might also decrease costs of ventilation [42,52], but this mechanism is not relevant for isopods, which ventilate passively. Importantly, in contrast to our findings in *P*. *scaber* woodlice, hypoxic conditions did not change the temperatures preferred by tarantula spiders [42]. This is somewhat surprising because in principle, arachnids and isopods have similar oxygen-delivery systems that involve a gas-exchange organ that exchanges gases with the ambient air (book lungs in spiders and pleopodal lungs in isopods) and a circulating haemolymph system that delivers oxygen to tissue with the help of oxygen-binding protein. On the other hand, isopods and spiders are distantly related groups, and many other mechanisms could account for this difference in the response to hypoxia.

We found a negative effect of hypoxia on the CT_max_ of *P*. *scaber* (measured in two different ways: heat coma and survival at high temperatures), which indicates that the upper thermal limits of terrestrial crustaceans can change with oxygen conditions in the environment. The two-stage gas-exchange system of isopods might be expected to increase the hypoxia sensitivity of thermal performance compared to the one-stage tracheal system of insects [30] because the affinity of haemocyanin to oxygen decreases with an increase in temperature [44]. The tracheal system delivers oxygen directly to insect tissues and lacks specialized carriers of oxygen [10], but binding proteins are used by some insects for oxygen storage [53]. Klok et al. [30] and Stevens et al. [31] compared the CT_max_ of isopods and insects by measuring the metabolic rate and examining their activity. In both studies, isopods (either *P*. *scaber* or *Armadillidium vulgare*) decreased their CT_max_ under hypoxia, in agreement with our results. Klok et al. [30] also showed that a beetle (*Gonocephalum simplex*) tolerated hypoxia well and did not change its CT_max_ (activity was decreased by only a decrease in the O_2_ level to 2.5%). Stevens et al. [31] reported that under hypoxia, a beetle (*Tenebrio molitor*) exhibited a decrease in CT_max_ of 6.9°C compared to an isopod, which decreased its CT_max_ by 10.6°C. Importantly, Stevens et al. [31] also examined the effects of hypoxia on cold tolerance (CT_min_), concluding that cold tolerance was affected by oxygen conditions in neither isopods nor insects. This conclusion also agrees with our evidence for *P*. *scaber*, which shows no effect of oxygen conditions on the tolerance of low temperatures. Oxygen limitation likely decreases in the cold as a result of a dramatic decrease in the rate of metabolism and thus in the demand for oxygen.

In the same thermal habitat, our experimental woodlice were able to run at a maximum of ca. 1.5 times faster under normoxia than under hypoxia, which suggests that the ability of *P*. *scaber* to perform highly metabolically demanding tasks can be limited by the oxygen supply in the air. The temperature at which the speed reached its maximum was lower under hypoxia (25.13°C) than under normoxia (28.87°C), although this difference was only nearly statistically significant. Thermal performance curves have rarely been studied in isopods, especially in the context of oxygen limitation, so we do not have many empirical references for evaluating whether this pattern is generally observed in *P*. *scaber* or other isopods. The evidence available in the literature indicates that running isopods achieve their highest speed at temperatures higher than those that maximized running speed in our experiment (ca. 25-29°C). For example, Dailey et al. [35] observed a continuous increase in the running speed of *Porcellio laevis* at temperatures approaching 35°C, but temperatures higher than 35°C were not tested; therefore, it is difficult to conclude the exact temperatures that maximize the performance of this species. Schuler et al. [54] observed that *P*. *scaber* reached its maximum speed in the range of 33-34°C, which was much higher than the temperature in our running essays. Certainly, the results of comparisons of nominal trait values between studies that involved different populations and methods should be interpreted with caution. For example, we forced woodlice to run for 15 minutes or until complete exhaustion, but the previous studies used much shorter observation times. Therefore, it is likely that instantaneous performance is maximized by higher body temperatures than is sustainable performance. If so, our results suggest that hypoxic conditions are the limiting factor for isopods involved in prolonged locomotor activity, but future studies should evaluate whether their instantaneous performance is also oxygen-sensitive.

Mobility has different selective advantages, including the capacity for behavioural thermoregulation, foraging, and predation avoidance [35]. Is not clear how well the ability of isopods to achieve high running speeds correlates with their predation avoidance or with other fitness-related consequences. According to Sunderland et al. [55], isopods might rarely be attacked by predators, and most such attacks are directed towards juveniles. However, some predators are believed to be isopod specialists, such as the woodlouse spider *Dysdera crocata* (but see [56]). The preferred temperatures of *D*. *crocata* [57] appear to closely match the preferred temperatures of its potential prey, the sympatric woodlouse *P*. *laevis* [58]. Other studies demonstrated that the presence of predator cues increased turn alternations in isopods [59]. Moreover, if spiders prove to be less oxygen-limited than isopods (as speculated in the first paragraph of our Discussion), then woodlouse spiders would not change their predation intensity on isopods in oxygen-deficient microhabitats, which would result in increased predation pressure on the oxygen-limited woodlice, causing them to become easy prey in hypoxic habitats. This scenario certainly requires rigorous testing.

Integrating our data on thermal preferences and performance, we found a notable mismatch between the temperatures preferred by woodlice and the temperatures at which woodlice achieved their peak locomotor performance: the preferred temperature was lower than the peak-performance temperature. Such mismatches might indicate a conflict between the habitat preferences of resting organisms that must involve decisions about energy expenditure and long-term fitness consequences and the thermal sensitivity of active organisms that must involve the thermal dependence of muscle physiology. Interestingly, the mismatch between the preferred and peak-performance temperatures was slightly larger under normoxia (a difference of 7.94°C) than under hypoxia (a difference of 7.45°C). Note here that the preferred temperatures were more strongly affected by oxygen availability than were the temperatures that ensured peak performance. According to Pörtner [60], aerobic scope (the difference between minimal and maximal metabolism) is a factor that integrates many physiological and ecological processes (immunological, behavioural, growth, foraging, etc.). Aerobic scope is expected to reach its maximum at an optimal temperature, decrease towards the thermal limits and approach zero at critical temperatures. Thus, a single optimal temperature is predicted for all physiological processes (the model described with reference to fish) [60,61]. Our evidence for the mismatch between the preferred temperatures and peak-performance temperatures is not consistent with such a single optimum temperature. Clark et al. [61] suggested that different types of performance should follow different thermal sensitivities with different optima rather than one universal pattern. From a broader life-history perspective, it is hard to imagine that the temperatures that maximize a given type of physiological performance are evolutionarily adaptive (e.g., an ectotherm that maintains a body temperature that maximizes locomotor performance does not necessarily maximize its expected lifetime reproductive output) only because different measures of performance and fitness play out on different timescales.

Overall, the results of our study provide important insight into the ecologically relevant consequences of micro-environmental gradients in temperature and oxygen availability. Importantly, we focused on multiple elements of thermal performance (thermal preferences, thermal limits, and thermal sensitivity of mobility), which helped us develop an integrated view of how differences in microhabitat temperature and oxygen availability might affect terrestrial isopods in nature. The detected mismatch between the temperatures that were preferred by woodlice and the peak-performance temperatures indicates that attempts to draw simplifying inferences about species-specific ecological optima should be made with caution. From a larger perspective, our results can help address how the oxygen sensitivity of thermal performance shapes the geographic distribution of terrestrial isopods and their expected responses to global climate change. It was already demonstrated that *P*. *laevis* adapts to local thermal conditions along latitudes, showing sharp latitudinal clines in thermal optima, thermal performance and thermal tolerance [58]. Additionally, species of isopods have evolved different respiratory organ anatomies, which likely reflect specializations to conditions of varying humidity [62,63]; however, these adaptations should also have consequences for oxygen deprivation tolerance [10].

## Supporting information

S1 Fig Distribution of randomly generated differences in the temperature at which the maximal performance (running speed) of common rough woodlice (*Porcellio scaber*) was achieved (T_MP_) between two oxygen treatments (normoxia and hypoxia). The distribution was obtained via 10000 randomizations (see Material and methods). The empirical difference in T_MP_ calculated from the original data is indicated by the red line.
(TIF)

S2 Fig Distribution of randomly generated differences in the maximal performance (MP, measured as running speed) of common rough woodlice (*Porcellio scaber*) between two oxygen treatments (normoxia and hypoxia). The distribution was obtained via 10000 randomizations as described in detail in the Material and methods section. The empirical difference in MP calculated from the original data is indicated by the blue line.
(TIF)
