# Supplementary figures and images for: Hypoxia causes woodlice (Porcellio scaber) to select lower temperatures and impairs their thermal performance and heat tolerance
Source: PLoS One. 2019 Aug 1;14(8):e0220647. doi: 10.1371/journal.pone.0220647 (PMC6675064; doi:10.1371/journal.pone.0220647)

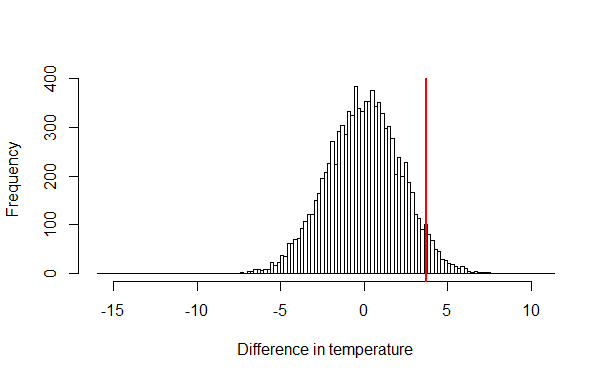

Supplement: S1 Fig — The distribution was obtained via 10000 randomizations (see Material and methods). The empirical difference in TMP calculated from the original data is indicated by the red line. (TIF) [file pone.0220647.s001.tif]

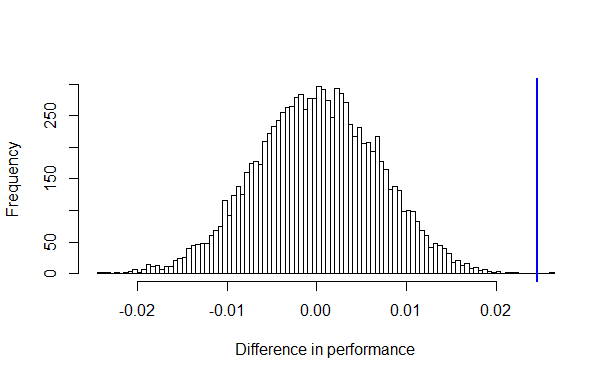

Supplement: S2 Fig — The distribution was obtained via 10000 randomizations as described in detail in the Material and methods section. The empirical difference in MP calculated from the original data is indicated by the blue line. (TIF) [file pone.0220647.s002.tif]
